# Cerebral Venous Sinus Thrombosis due to Iron Deficiency Anemia in an Adolescent Girl

**DOI:** 10.1155/2021/9979078

**Published:** 2021-07-19

**Authors:** Hussein N. Matlik, Nivargi Sagar

**Affiliations:** Burjeel Hospital, Abu Dhabi, UAE

## Abstract

Cerebral venous sinus thrombosis (CVST) is rare but now increasingly diagnosed in children. Early diagnosis is of prime importance as any delay leads to significant mortality and morbidity. It requires a high index of suspicion to diagnose CVST early as, often, the symptoms are vague and the signs are nonspecific. Varieties of aetiologies are described for generation of cerebral venous sinus thrombus. Iron deficiency anemia is one of the most important preventable causes of CVST. The most proposed mechanism in development of CVST in iron deficiency is secondary thrombocytosis. However, we describe a case of CVST due to iron deficiency in the absence of thrombocytosis.

## 1. Introduction

Cerebral venous sinus thrombosis is an uncommon diagnosis in children; however, it is more frequently diagnosed now with improving awareness in physicians, efficient imaging techniques, and survivors of previously fatal diseases that predispose to development of CVST. Most of the literature on childhood CVST comprises of case reports or small case series owing to small number of cases diagnosed. The incidence of CVST was 0.67 cases per 100,000 children per year in one case series [1]. Similar to adults, CVST in children is multifactorial comprising of an exhaustive list: thrombophillic states (protein C deficiency, protein S deficiency, Factor V Leiden, prothrombin gene mutation, antithrombin III deficiency, etc.), childhood infections (meningitis, otitis media, mastoiditis, etc.), dehydration, intracranial surgeries, hematological diseases (anemia, sickle cell disease, thalassemia major, etc.), and systemic illness such as nephrotic syndrome, systemic lupus erythematosus, and congenital heart diseases to name a few. In most of the pediatric patients, the presenting symptoms of CVST are often nonspecific and include headache, lethargy, vomiting, altered mental status, focal neurodeficits, and seizures (more common manifestation in newborns with CVST). We present here a 15-year-old adolescent girl with CVST who presented with persistent headache.

## 2. Case Report

**Figure 1:**
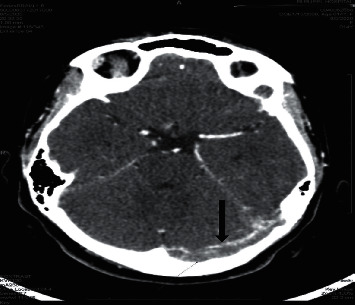
Plain CT scan axial cut shows relative density along the left transverse sinus (arrow).

**Figure 2:**
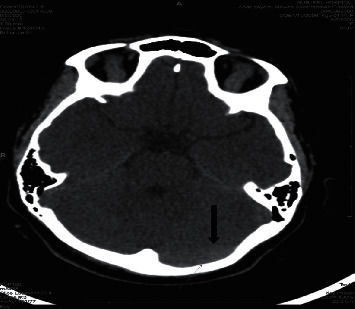
Postcontrast CT axial cut shows filling defect in the transverse sinus suggesting thrombosis (arrow).

**Figure 3:**
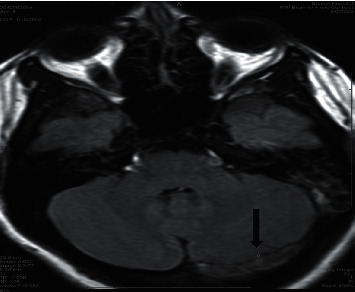
MRI axial flair images show altered SI of the left transverse sinus (arrow).

**Figure 4:**
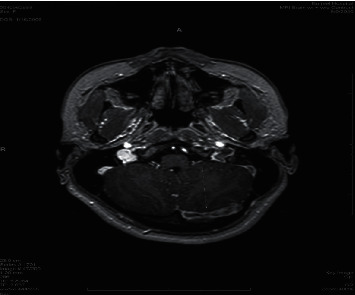
Postcontrast MRI axial cuts show filling defect in the left transverse sinus (arrow).

**Figure 5:**
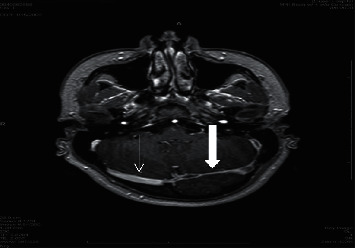
Postcontrast MRI axial cuts show filling defect in the left transverse sinus (bold arrow) and normal contrast filling (arrow) in the right transverse sinus.

**Figure 6:**
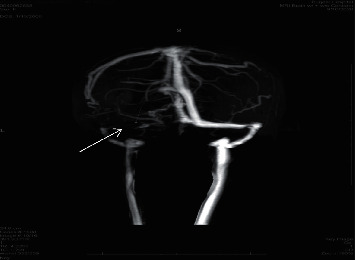
MR venography shows filling defect attenuating the left transverse sinus and sigmoid and internal jugular veins (arrow).

A previously healthy 15-year-old adolescent girl presented with complaints of episodic headache for 1 week. Headache was neither associated with nausea, vomiting, coryzal symptoms, cough, and fever nor had any aggravating or relieving factor. There were no abnormal physical findings at the first examination. Hence, oral analgesic was prescribed. Later, she followed up in the Emergency Department with persistent headache now associated with vomiting and dizziness. On examination, she had significant pallor and moderate dehydration but she maintained acceptable peripheral perfusion. The rest of the physical examination was unremarkable without any signs of raised intracranial pressure (ICP) or any neurodeficits. The investigations revealed severe microcytic hypochromic anemia (Hb) 7.7 g/dL (12–15), MCV 62.30 fl (81–99), MCH 16.70 pg (27–32), MCH 26.70 g/dL (32–36), high red cell distribution width (RDW) 20.30% (11.6–14), and low ferritin 4.66 *µ*g/L (13–68). In view of persistent headache, neuroimaging was ordered. Computed Tomography (CT) head showed extensive thrombus in the straight and left transverse sinus extending to the left sigmoid sinus and internal jugular vein (IJV) (Figures 1 and 2). This was confirmed on magnetic resonance imaging (MRI) (Figures 3–5) and MR venography (Figure 6). There was no evidence of cerebral hypoperfusion or infarction. She was admitted to the pediatric intensive care unit (PICU). Paracetamol was used for analgesia along with supportive neuroprotective measures. Low-molecular-weight heparin (enoxaparin subcutaneous 1 mg/kg/dose twice daily) was started upon the diagnosis of CVST. With IV rehydration and supportive care, her general condition improved. She remained neurologically and hemodynamically stable and, hence, was shifted to the inpatient unit. She developed bilateral papilledema and right abducent nerve paresis (right cranial nerve VI) on the 2^nd^ day of admission. Oral analgesic was continued, and oral acetazolamide was added. She responded well to acetazolamide and oral paracetamol as her headache reduced and movement of right lateral rectus improved too. Oral iron was prescribed for the iron deficiency. She was discharged home on the 5^th^ day. A week later, she was examined in the outpatient clinic where she had complete resolution of headache, diplopia, right CN VI paresis, and papilledema. Anticoagulation was monitored with activated factor X level. Acetazolamide was stopped then. LMW heparin was prescribed for 3 months.

Prothrombin time/International Normalized Ratio (PT/INR) and activated partial thromboplastin time (aPTT) was normal. Sickle cell screening was negative. Platelet count was normal. Thrombophilia work-up showed normal level of homocysteine, protein C (98.7%), protein S (87.8%), antithrombin III (109.5%) activities, and lipoprotein A. She tested negative for prothrombin gene mutation (20210 gene mutation), Factor V Leiden, antinuclear and anti-double-stranded DNA antibodies, and antiphospholipid and anticardiolipin antibodies.

## 3. Discussion

Cerebral venous sinus thrombosis (CVST) is occlusion of drainage of the intracranial venous sinuses and cerebral veins by a thrombus leading to the development of intracranial hypertension and/or venous infarcts. This leads to significant mortality and morbidity due to long-term neurological sequelae unless diagnosed at an early stage.

In the largest case series from Canada, the incidence noted is 0.67 cases per 100000 children per year with infants less than one year being affected the most [1]. The disorder is more frequently diagnosed now with advances in neuroimaging modalities; however, the incidence might be reducing as our understanding of etiopathogenesis of CVST is increasing and the incidence of some of the underlying predisposing conditions is decreasing such as congenital heart diseases and head and neck infections and middle ear infections.

The symptoms and signs are often vague leading to a delayed diagnosis. Symptoms can vary from diffuse neurologic such as persistent headache, vomiting, and altered mental status to focal such as seizures and neurodeficits. Seizure as a manifestation of CVST is more likely in neonates, while older children present more likely with headache, vomiting, and altered mentation [2].

CVST has multiple etiological factors. A significant proportion of children have an underlying thrombophilic disorder [3–5]. However, it is postulated that there are several “triggers” or prothrombotic risk factors that induce and propagate CVST. Local or systemic infection, trauma, hematological disorders such as anemia, underlying malignancy with or without chemotherapeutic drugs, congenital cyanotic heart disease, nephrotic syndrome, and connective tissue disorders such as systemic lupus erythematosus are a few of the prothrombotic risk factors worth a mention [3–6].

Anemia is a well-known prothrombotic risk factor. Chronic hemolytic anemias such as sickle cell anemia have a well-described association with arterial strokes. However, iron deficiency anemia alone has been implicated in causing CVST in multiple studies [7–12]. Iron deficiency anemia has alone been a risk factor for stroke [13].

Iron is a key regulator of thrombopoiesis as normal iron level is essential to inhibit thrombopoiesis. Iron deficiency induces secondary thrombocytosis [14]. Thrombocytosis increases the blood viscosity, thus predisposing to thrombosis.

Iron deficient microcytes are less deformable compared to normocytes, thus creating a turbulent flow. Turbulent blood is an independent risk factor for generation and propagation of thrombus.

Iron deficiency anemia hampers oxygen delivery to end organs. Cerebral circulation compensates this by increasing cerebral blood flow. Thus, the probability of CVST exponentially increases in a turbulent cerebral blood flow.

CVST is a diagnosed by neuroimaging, magnetic resonance imaging (MRI) with venography (MRV) being the preferred diagnostic modality. Venography helps locating the thrombus and shows its extent as well while MR imaging shows venous ischaemia if any [1, 13]. Follow-up imaging is required for diagnosing resolution of the thrombus and recanalization of the venous sinuses. The next step is to look for a prothrombotic disorder. At least, one prothrombotic factor is found in 38% children while two or more factors are documented in 65% cases [3–5]. However, in one-third patients, no obvious cause is found [15].

Currently, there are no pediatric trials addressing the use of anticoagulant therapy. The use of anticoagulation is based on its obvious benefits in adult trials. Kenet et al. mentioned nonadministration of anticoagulant before relapse as an independent risk factor for recurrent CVST [16]. Most of the centers are against the use of anticoagulation in neonates owing to the risk of hemorrhage. However, most of the younger children receive an anticoagulant. Most used anticoagulation is low-molecular-weight heparin. A strict therapeutic monitoring is required using activated Factor X level. Usual duration of therapy is 3–6 months. Periodic MRI and MRV are recommended and are usually performed at an interval of 3 months for 1 year. It helps not only to monitor recanalization of the sinuses but can also show progression of thrombus. In a European study, complete and partial recanalization was observed in 46% and 42%, respectively, in 3–6 months. The same study mentions an early mortality around 3% and recurrence rate of 6%. Most of the recurrent episodes (70%) were observed within 6 months of the initial event [16].

## 4. Conclusions

CVST is an uncommon diagnosis in children that presents with nonspecific symptoms mandating high risk of suspicion for early diagnosis. Apart from prothrombotic disorders, iron deficiency is a less recognized predisposing factor although highly prevalent in children. Treatment of iron deficiency has been recommended for a variety of reasons, but this case report provides another important but less-known reason to treat iron deficiency.
